# Fetal Ductal Constriction, Pulmonary Hypertension and Atrial Septum Mobility: A Correlation Study

**DOI:** 10.1002/jum.70130

**Published:** 2025-11-20

**Authors:** Paulo Zielinsky, Polyanna Henriques, Pedro Van Der Sand, Maria Antonia Saldanha, Gabriela Macelaro, Joana Nicoloso

**Affiliations:** ^1^ Fetal Cardiology Unit Instituto de Cardiologia/Fundação Universitária de Cardiologia—IC/FUC Porto Alegre RS Brazil; ^2^ Department of Pediatrics Universidade Federal do Rio Grande do Sul—UFRGS Porto Alegre RS Brazil

**Keywords:** duct constriction, fetal echocardiography, prostaglandin inhibitors, septum primum excursion index

## Abstract

**Objectives:**

Fetuses with ductal constriction (FDC), pulmonary hypertension and right ventricular overload after maternal exposure to PGE2 inhibitors—nonsteroidal anti‐inflammatory drugs (NSAIDs) or polyphenols (PF)—have increased septum primum excursion index (SPEI), the ratio between maximal septum primum displacement and left atrial diameter. The objective of this study is to assess SPEI behavior and its correlation to mean pulmonary artery pressure (MPAP) after ductal constriction (DC) resolution.

**Methods:**

Cohort study comparing SPEI and MPAP during and 2 weeks after DC reversal, following discontinuation of NSAIDs and PF. Criteria for DC diagnosis were systolic velocity >1.40 m/second, diastolic velocity >0.30 m/second, and pulsatility index <2.2. MPAP was estimated by Dabestani equation: MPAP = 90 − (0.62 × pulmonary artery acceleration time). Statistical analysis: *t*‐test and Pearson's correlation.

**Results:**

Fifty‐two pregnant women with FDC were evaluated. Following reversal, mean PI increased from 1.89 ± 0.20 to 2.54 ± 0.27 (*p* < .001), mean SPEI decreased from 0.75 ± 0.13 to 0.42 ± 0.12 (*p* < .001), and MPAP decreased from 70.33 ± 5.52 mmHg to 53.27 ± 6.68 mmHg (*p* < .001), with a significant correlation between MPAP and SPEI (*r* = 0.690).

**Conclusion:**

After resolution of fetal DC, the SPEI decreases, this effect being correlated with reduction in MPAP.

**Abbreviations**
DC: ductal constriction
DV: diastolic velocity
FDC: fetuses with ductal constriction
GA: gestational age
LA: left atrium
MPAP: mean pulmonary artery pressure
NSAID: nonsteroidal anti‐inflammatory drug
PF: polyphenol
PI: pulsatility index
RA: right atrium
SPEI: septum primum excursion index
SV: systolic velocity

Fetal ductal constriction (DC), induced by maternal use of prostaglandin E2 inhibitors such as nonsteroidal anti‐inflammatory drugs (NSAIDs)^1^, ^2^, ^3^, ^4^, ^5^ and polyphenols, especially^6^, ^7^, ^8^, ^9^ in the third trimester of pregnancy—poses a serious risk to fetal health. These substances interfere with the synthesis of prostaglandins, which are essential for maintaining the patency of the ductus arteriosus, a vital connection between the pulmonary artery and the aorta during fetal life.

The action of prostaglandin E2 inhibitors may lead to premature closure of the ductus arteriosus, impairing normal blood flow and compromising fetal circulation, with well‐known complications. DC can lead to fetal pulmonary hypertension, a condition characterized by elevated pressure in the pulmonary circulation due to increased pulmonary vascular resistance.^10^, ^11^, ^12^, ^13^, ^14^, ^15^, ^16^, ^17^, ^18^, ^19^

The main functional consequence of DC is right ventricular overload. The elevated pulmonary arterial pressure increases the workload of the right ventricle, potentially leading to right ventricular hypertrophy and, in severe cases, right heart failure. This can result in decreased perfusion to vital fetal organs, with a risk of hypoxia and other forms of tissue injury.^18^ DC caused by these agents is usually reversible upon discontinuation of prostaglandin E2 inhibitors. The condition typically resolves within 2 weeks, with regression of DC, restoration of adequate blood flow, and improvement in both pulmonary hypertension and right ventricular overload.^19^, ^20^

The movement of the septum primum depends on fetal cardiac function.^21^, ^22^ When the right ventricle is overloaded due to pulmonary hypertension, elevated right intraventricular pressure leads to increased right atrial pressure, which in turn increases septum primum excursion—as previously demonstrated.^23^

The conceptual hypothesis of the present study was that in fetuses with ductal constriction (FDC), where the septum primum excursion index (SPEI) is increased, this index would decrease following resolution of the constriction, and that this decrease would correlate with a reduction in mean pulmonary artery pressure (MPAP).

## Methods

### Study Design and Population

This prospective cohort study evaluated echocardiographic measurements in fetuses with a gestational age (GA) of 27 weeks or more, both at the time of diagnosis of DC and after a 2‐week intervention involving the suspension of NSAIDs and polyphenol‐rich foods. The Strobe Statement was followed.

Based on a pilot study that assessed the behavior of the SPEI in FDC and in healthy fetuses, a minimum sample size of 24 patients was calculated for a significance level of .05, accounting for possible losses and refusals.^23^

Fetuses were excluded if they had other functional or morphological cardiac abnormalities or if their mothers had risk factors for fetal heart disease or any condition that could interfere with echocardiographic imaging—such as gestational diabetes or hypertension. Fetuses with a GA below 27 weeks were also excluded. GA was confirmed using either the date of the last menstrual period or a first‐trimester ultrasound.

### Fetal Echocardiography

All pregnant participants underwent 2 echocardiographic evaluations: the first at the time of diagnosis and the second after a 2‐week period following the removal of ductal constrictive agents. The examinations were performed by 2 pediatric cardiologists experienced in fetal echocardiography, using General Electric Voluson E10, E8, and E6 ultrasound machines with 3.0–5.0 MHz convex transducers, configured for fetal cardiology.

**Figure 1:**
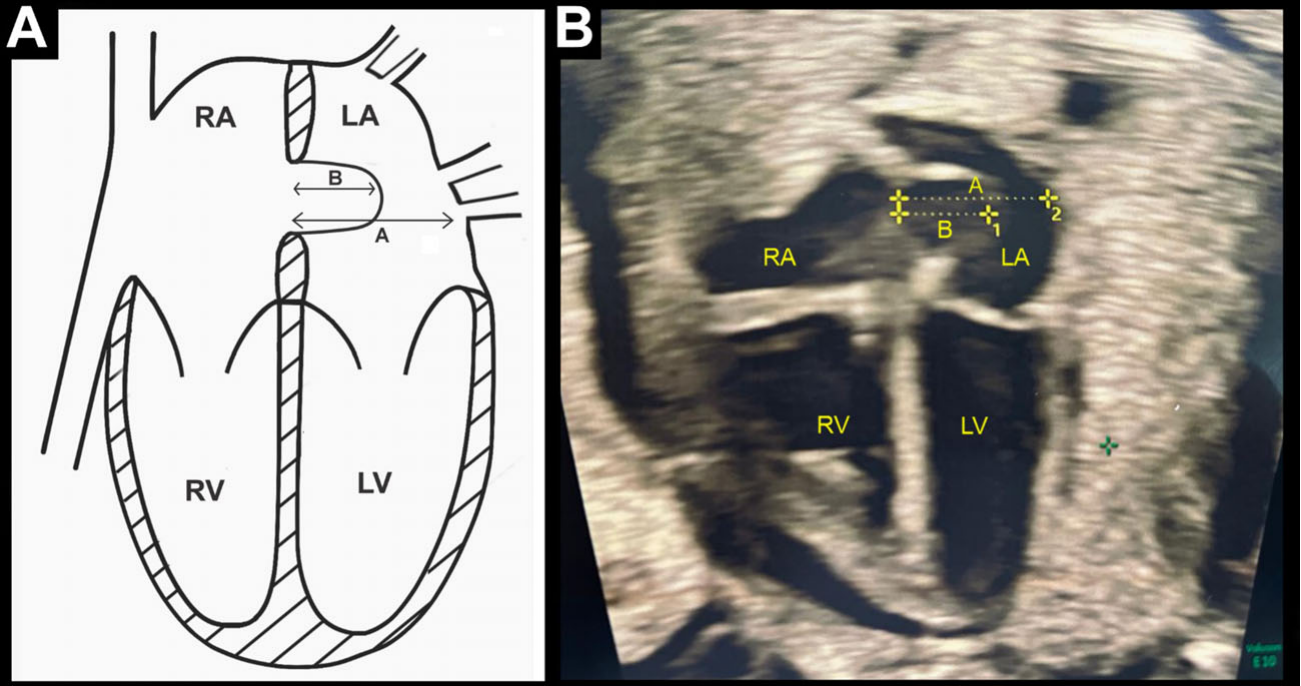
Fetal echocardiographic image of *septum primum* excursion. **A**, Schematic drawing of *septum primum* excursion. **B**, Still picture of fetal echocardiographic appearance of *septum primum* excursion.

Diagnosis of DC was based on the assessment of ductus arteriosus flow using the following criteria: turbulent ductal flow, peak systolic velocity (SV) ≥1.40 m/second, peak diastolic velocity (DV) ≥0.30 m/second, and a pulsatility index (PI) <2.2, calculated as (systolic velocity − diastolic velocity)/mean velocity.^6^, ^7^, ^8^ The SPEI was measured by 2D echocardiography as the ratio between the maximum linear displacement of the foramen ovale valve in diastole and left atrial diameter in the 4‐chamber view (Figure 1, A and B), as previously described.^21^, ^24^

MPAP was calculated using the Dabestani equation: **MPAP = 90 − (0.62 × pulmonary artery acceleration time).^10^, ^11^, ^12^, ^25^

### Statistical Analysis

Statistical analysis was performed using SPSS® software, version 24.0. Mean values of the variables were compared between the first (diagnosis) and second (post‐intervention) consultations using paired Student's *t*‐test. One patient who did not experience resolution of constriction was excluded from the analysis.

To assess the correlation between the dynamics of the SPEI and MPAP, Pearson's correlation coefficient was calculated, adjusted for GA. Continuous variables (GA, ductal PI, MPAP, and SPEI) were compared using Student's *t*‐test, with a significance level set at 5%, after confirming the normality of the sample with the Kolmogorov–Smirnov Test.

### Ethical Aspects

This study was conducted in accordance with the ethical guidelines established by the National Commission for Research Ethics (CONEP‐Brazil). The research protocol was previously approved by the Research Ethics Committee of IC/FUC – Ethics Approval number 5.050.303—CAAE 49753321.2.0000.5333.

All participants received detailed information about the objectives of the study and procedures. Those who agreed to participate signed an informed consent form, confirming their voluntary participation and their right to withdraw from the study at any time.

## Results

Fifty‐two pregnant women diagnosed with fetal DC were evaluated. Of these, 51 showed reversal of the condition following intervention; 1 patient, in whom reversal did not occur, was excluded from the final analysis.

Mean maternal age among participants was 31 ± 6 years. Mean GA at the time of the first evaluation was 30.6 ± 2.9 weeks, and at the follow‐up assessment it was 33.0 ± 0.10 weeks (*p* < .001).

**Figure 2:**
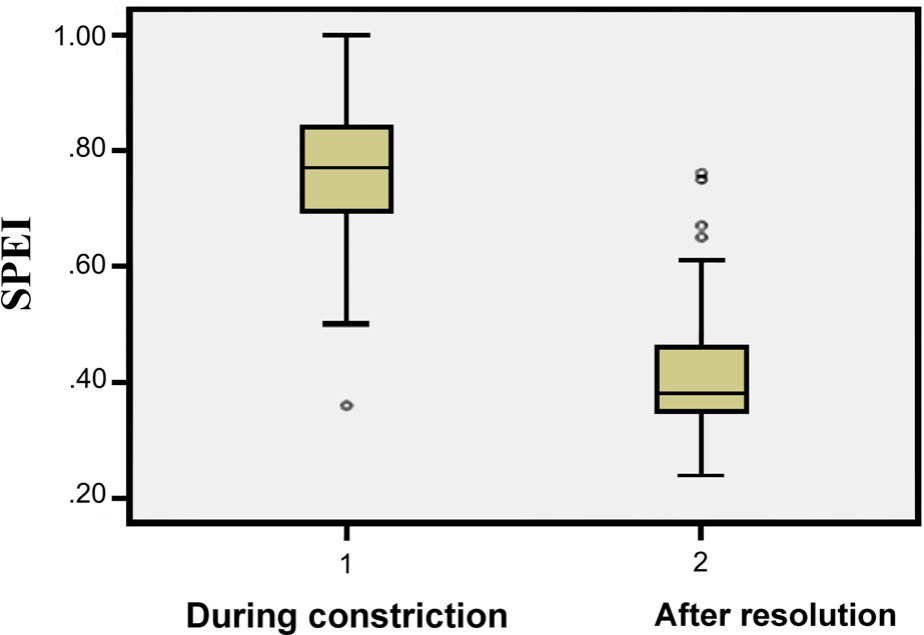
Before‐and‐after plot of *septum primum* excursion index during ductal constriction and after reversal.

**Figure 3:**
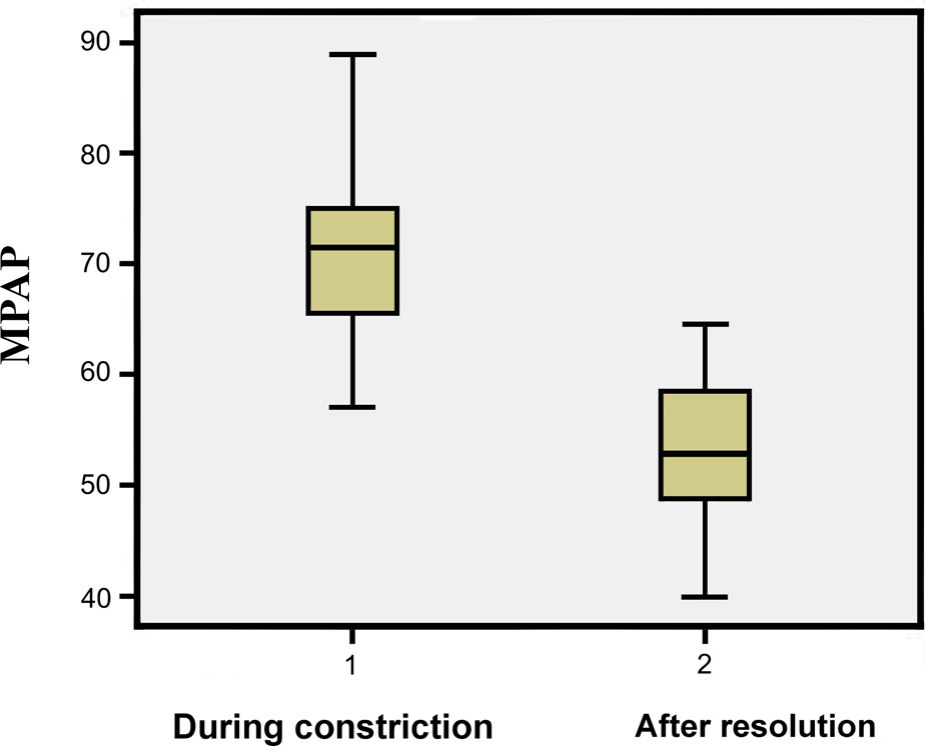
Before‐and‐after plot of fetal surrogate mean pulmonary artery pressure during ductal constriction and after reversal.

Mean PI increased significantly after resolution of DC, from 1.89 ± 0.20 to 2.54 ± 0.27 (*p* < .001). Mean SPEI decreased from 0.75 ± 0.13 (range: 0.36–1.02) to 0.42 ± 0.12 (range: 0.24–0.59) (*p* < .001) (Figure 2). Likewise, MPAP decreased from 70.33 ± 5.52 mmHg to 53.27 ± 6.68 mmHg (*p* < .001) (Figure 3).

**Figure 4:**
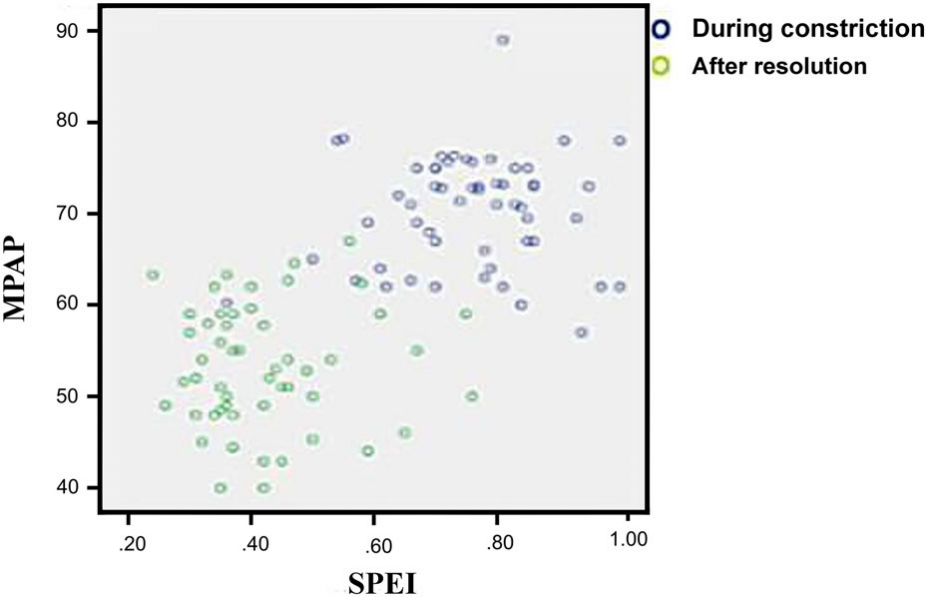
Correlation between *septum primum* excursion index and surrogate mean pulmonary artery pressure before and after reversal of ductal constriction.

A statistically significant positive correlation was observed between MPAP and SPEI, adjusted for GA (*r* = 0.690) (Figure 4).

## Discussion

This study demonstrates, for the first time, that the SPEI, which is increased during DC in third‐trimester fetuses,^25^ decreases following the resolution of this functional impairment. This decrease is correlated with a reduction in MPAP.

In the presence of DC, pulmonary hypertension is inherently present and represents the first and most significant consequence of elevated fetal pulmonary vascular resistance. Previous studies have shown that pulmonary hypertension that is secondary to DC improves after its resolution and that this improvement is followed by enhanced pulmonary vascular maturity.^9^, ^10^, ^11^, ^12^, ^13^, ^14^, ^15^, ^16^, ^17^, ^18^

Recognizing that the foramen ovale is the primary site of communication between the atria and understanding that the mobility of the septum primum reflects changes in atrial dynamics, we have previously demonstrated that fetuses^25^, ^26^ of diabetic mothers with secondary septal hypertrophy (and consequent impaired left ventricular compliance or relaxation) had reduced septum primum mobility during diastole, probably as a result of increased left atrial pressure. More recently we hypothesized, by analogy, that increased right intraventricular and atrial pressure—secondary to fetal pulmonary hypertension present in DC—could enhance this mobility, increasing the SPEI. This hypothesis was confirmed in a previous study^24^. The “excursion index” used to quantify septum primum movement was defined as the ratio between the maximum linear displacement of the septum primum at the end of diastole and the maximum left atrial diameter, obtained by 2‐dimensional fetal echocardiography in the 4‐chamber view.

Therefore, septum primum mobility, represented by its excursion index, is increased in third‐trimester FDC and reflects elevated pulmonary artery pressure. In the present study, we demonstrated that resolution of this condition—occurring 2–3 weeks after maternal suspension of NSAIDs or polyphenol‐rich foods—was associated with a reduction in septum primum excursion and that this reduction was strongly correlated with improvement in pulmonary hypertension.

Introducing this variable—SPEI—may provide valuable diagnostic and prognostic information. It has the potential to serve as another marker of right ventricular dysfunction and fetal pulmonary hypertension, which could guide clinical recommendations regarding the restriction of pharmacological or dietary agents implicated in the pathogenesis of DC.

As for limitations, we acknowledge the potential bias inherent in data collected from a single center. However, the reproducibility of the echocardiographic variables has been previously evaluated.^22^ The reliance on indirect parameters to assess surrogate pulmonary hypertension and DC also presents a potential limitation, though this is a common challenge in clinical studies within this field and has already been commented.^22^

As for limitations, we acknowledge the possible bias inherent of a monocentric study. However, the reproducibility of the echocardiographic variables has been previously evaluated.^22^ The reliance on indirect parameters to assess surrogate pulmonary hypertension and DC also presents a potential limitation, though this is a common challenge in clinical studies within this field and has already been addressed.^22^ The pressure in the right atrium (RA) during pregnancy is physiologically higher than in the left atrium (LA). Even though the enlargement of the RA can be observed in normal pregnancies, more often it is correlated with cardiac pathologies that involve an overload of the right ventricle or even genetic myopathies due to the presence of muscular tissue.^27^ Variability in RA volume and pressure could potentially interfere with SPEI.

Other possible interfering factors in the measurement of the SPEI could be the size of the foramen ovale which could be enlarged or restricted.^28^, ^29^ In the present study group, we excluded conditions of pathological enlargement of the RA and dimensions of the foramen ovale.

## Conclusion

In conclusion, third‐trimester FDC and an elevated SPEI exhibit a significant reduction of this index following resolution of constriction. This reduction is strongly correlated with improvement in intrauterine pulmonary hypertension.

These findings support the clinical value of the assessment of SPEI as a non‐invasive echocardiographic parameter that reflects changes in fetal pulmonary hemodynamics and may assist in monitoring the expected resolution of DC after recommendation of decreasing maternal ingestion of known prostaglandin inhibitors.

## Supporting information


**Data S1.** STROBE Statement—checklist of items that should be included in reports of observational studies.

## Data Availability

The data that support the findings of this study are available from the corresponding author upon reasonable request.
